# Effects of different vasopressors on the contraction of the superior mesenteric artery and uterine artery in rats during late pregnancy

**DOI:** 10.1186/s12871-021-01395-6

**Published:** 2021-06-30

**Authors:** Tingting Wang, Limei Liao, Xiaohui Tang, Bin Li, Shaoqiang Huang

**Affiliations:** 1 Department of Anaesthesia, Changning Maternity and Infant Health Hospital, Shanghai, 200050 China; 2 Department of Anesthesiology and Perioperative Medicine, Guangzhou Women and Children’s Medical Center, Guangzhou Medical University, 9# Jinsui Road, Guangzhou, 510623 China; 3 Department of Anaesthesia, Obstetrics & Gynecology Hospital, Fudan University, 128# Shenyang road, Shanghai, 200090 China

**Keywords:** Vasopressor, Mesenteric artery, Uterine artery, Contraction, Caesarean section

## Abstract

**Background:**

Hypotension after neuraxial anaesthesia is one of the most common complications during caesarean section. Vasopressors are the most effective method to improve hypotension, but which of these drugs is best for caesarean section is not clear. We assessed the effects of vasopressors on the contractile response of uterine arteries and superior mesenteric arteries in pregnant rats to identify a drug that increases the blood pressure of the systemic circulation while minimally affecting the uterine and placental circulation.

**Methods:**

Isolated ring segments from the uterine and superior mesenteric arteries of pregnant rats were mounted in organ baths, and the contractile responses to several vasopressor agents were studied. Concentration-response curves for norepinephrine, phenylephrine, metaraminol and vasopressin were constructed.

**Results:**

The contractile response of the mesenteric artery to norepinephrine, as measured by the pEC50 of the drug, was stronger than the uterine artery (5.617 ± 0.11 vs. 4.493 ± 1.35, *p* = 0.009), and the contractile response of the uterine artery to metaraminol was stronger than the mesenteric artery (pEC50: 5.084 ± 0.17 vs. 4.92 ± 0.10, *p* = 0.007). There was no statistically significant difference in the pEC50 of phenylephrine or vasopressin between the two blood vessels.

**Conclusions:**

In vitro experiments showed that norepinephrine contracts peripheral blood vessels more strongly and had the least effect on uterine artery contraction. These findings support the use of norepinephrine in mothers between the time of neuraxial anaesthesia and the delivery of the foetus.

## Background

Hypotension after neuraxial anaesthesia is one of the most common complications during caesarean section. Without preventive treatment, the incidence of hypotension during caesarean section is as high as 80% [1]. Severe hypotension causes nausea, vomiting, dizziness, confusion, and cardiac arrest.

Methods to prevent and treat hypotension during caesarean section include volume supplementation before anaesthesia, reduction of anaesthetic doses and injection speeds, post-anaesthesia position adjustment and vasopressor use. The use of vasopressor agents is the most effective method to improve hypotension [2]. Vasopressors that are commonly administered to pregnant women in clinical practice include phenylephrine, norepinephrine, metaraminol, and vasopressin. Each of these agents has its own advantages and disadvantages, but the existing research is limited to the effects of vasopressors on maternal circulation changes and neonatal acidaemia. Whether vasopressors affect perfusion of the largest visceral blood vessel affecting the foetus, i.e., the uterine artery, is not clear. The ideal vasopressor should effectively contract the peripheral blood vessels during after neuraxial anaesthesia and before the delivery of the foetus.

Phenylephrine, norepinephrine and metaraminol stimulate adrenaline receptors, and vasopressin increases blood pressure by stimulating V receptors on the cell membrane. However, the distribution and density of adrenaline and V receptor subtypes vary between different types of vascular beds, which cause different blood vessels to exhibit different vasoconstrictive responses to the same agents [3]. Changes in hormone levels during pregnancy may also affect the vasoconstrictive response to vasopressors. For example, Colucci et al. extracted blood vessels from male rats treated with 17β-oestradiol and found that the contractile response of the mesenteric arteries to norepinephrine was 4 times lower than non-oestradiol-treated rats [4]. Magness et al. measured uterine blood flow and uterine vascular resistance of sheep after a bolus of norepinephrine and phenylephrine and found that the effects of α-adrenergic agonists on the uterine vascular system were weakened during pregnancy [5]. These studies suggest that the effects of various vasopressors on the uterine arteries and the smooth muscles of the systemic circulation differ between pregnant and non-pregnant states.

The relative efficacy of blood vessels is particularly related to the selection and dose of appropriate vasopressors, and different blood vessels may respond differently to the vasopressor. However, a current limitation on research in this area is that the extraction of human blood vessels is subject to many ethical restrictions. We used isolated blood vessels of rats to simulate human vessels because of the 90% genetic similarity between rats and humans. In this study, we compared the effects of different vasopressors on the contractile response of uterine arteries and superior mesenteric arteries in pregnant rats, discussed the clinical implications of the vasopressor responses of isolated arteries in order to provide a basis for choosing the best vasopressor during the period between neuraxial anaesthesia and delivery of the foetus.

## Methods

### Laboratory animals

All animal experiments were performed in accordance with the basic principles of Fudan University’s animal experiments. The Animal Ethics Committee of Shanghai Medical College of Fudan University approved the protocol (201907007Z), and specific-pathogen-free (SPF) Sprague-Dawley rats (SD) were selected and purchased from Shanghai Jie Sijie Laboratory Animal Co., Ltd.

Female rats over 8 weeks of age with a weight of 180–200 g and fertile male rats weighing over 250 g were combined in a 2:1 ratio, and the bottom plate under the rearing cage was washed and covered with white paper. After successful mating, a light yellow cone was observed at the vaginal opening of the female rats, which is formed by coagulation of the semen secretion of the male rat. The vaginal plug is excreted after a few hours. The appearance of a vaginal plug the next morning was considered a successful pregnancy and recorded as the first day of pregnancy.

Pregnant females were placed in individual cages under a 12:12-h light-dark cycle. The rats had free access to standard food and tap water. Pregnant rats were used in experiments after 20 days of pregnancy.

### Superior mesenteric artery and uterine artery acquisition

Animals were euthanized using sodium pentobarbital (100 mg/kg, IP). The abdominal cavity was exposed via a midline incision. An intestinal segment from the beginning of the duodenum to the end of the ileum was taken along with its mesangium, the uterine tissue and the communicating blood vessels between these tissues. Two aliquots of physiological salt solution (PSS) were prepared, precooled to 4 °C, and fully oxygenated. The PSS consisted of the following components (in mmol): NaCl, 118.3; KCl, 4.7; MgSO_4_, 1.2; KH_2_PO_4_, 1.22; CaCl_2_, 2.5; NaHCO_3_, 25.0; calcium ethylenediaminetetraacetate, 0.016; and glucose, 11.1.

The adipose tissue, connective tissue around the selected secondary/tertiary artery and the veins accompanying the artery were removed under a dissecting microscope. A pathologist separated clean superior mesenteric arteries, which were trimmed into two 3-mm-long vascular rings that were threaded and fixed in a vascular tone measurement system [DMT 620 M vascular tone measurement system (Denmark)]. Fixed rings were filled with 5 ml of PSS solution at a constant temperature of 37 °C, and the solution was bubbled with 95% oxygen and 5% carbon dioxide. Care was taken throughout the process to avoid damaging the vascular endothelium.

The fourth-order branches (*0.8 mm in external diameter) of the main uterine arteries were separated from the surrounding tissue and cut into 3-mm ring segments. The small branches of the uterine arteries were chosen because they are similar in nature to arterioles and play a substantial role in vascular resistance. The remaining steps were the same as the superior mesenteric artery.

### Measurement of vascular ring tension

The transmural pressure of the superior mesenteric artery and uterine artery was adjusted to balance under a basal tension of 3 mN for 1 h. After calibration to zero, potassium chloride (KCl) was administered twice to stimulate vasoconstriction. Each stimulation used 60 mM KCl and lasted 10 min. After the contraction tension stabilized, the average contraction strength was recorded and calculated (i.e., ΔKCl = Ʃ(peak contraction response - base tension value before contraction)/2). The arteries were rinsed with PSS 3 times for 10 min each to restore the contraction curve to baseline levels.

The integrity of vascular endothelial function was tested by the addition of gradually increasing concentrations of phenylephrine (PE, 10^− 9^ to 10^− 4^ M) into the bath of the vascular tension measurement system to cause maximum contraction and the addition of gradually increasing concentrations of acetylcholine (ACh, 10^− 9^ to 10^− 4^ M) to reduce the vascular tension to the lowest value and stabilized. Endothelial function was considered lost when the decrease in vascular tension was less than 10% of the maximum contraction caused by PE [6]. Endothelial function was considered intact when the decrease in vascular tension was greater than 80% of the maximum contraction caused by PE [6]. The endothelium was considered damaged when the vascular tension dropped between 10 and 80% [6]. Only vascular rings with an intact endothelium were used in these experiments.

**Fig. 1:**
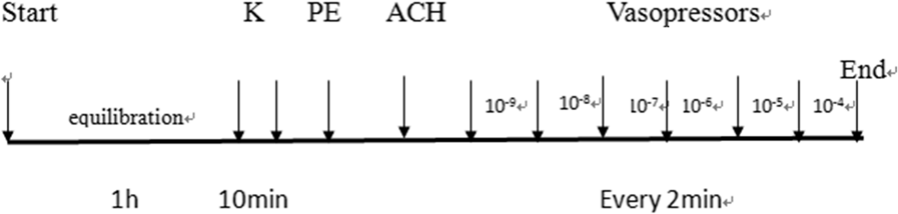
Experimental design. The transmural pressure of the superior mesenteric artery and uterine artery was adjusted to balance under a basal tension of 3 mN for 1 h. After zero adjustment, 60 mM potassium chloride (KCl) was used twice to stimulate vasoconstriction for 10 min. The integrity of vascular endothelial function was tested (phenylephrine, PE, 10^− 9^ to 10^− 4^ M; acetylcholine, ACH, 10^− 9^ to 10^− 4^ M). Gradually increasing concentrations of vasopressors (phenylephrine, norepinephrine, vasopressin, metaraminol) were added to the water bath (10^− 9^–10^− 5^/10^− 4^ M). Each drug concentration acted for 2 min

After testing the integrity of arterial endothelial function, we waited for the vascular tension curve to stabilize at the baseline level and added gradually increasing concentrations of vasopressors (phenylephrine, norepinephrine, vasopressin, metaraminol) to the water bath (10^− 9^–10^− 5^/10^− 4^ M). Each drug concentration was allowed to reach a stable level for 2 min to ensure that the vascular tension reached a stable level. The contraction strength was observed and recorded at each concentration (i.e., Δcontraction strength = peak contraction response - baseline tension value). After stimulation with the highest drug concentration, the vascular ring was washed twice with PSS solution for 5 min each, and the activity of the vascular ring was measured during stimulation with 60 mM KCl (Fig. 1).

Vascular rings that did not respond to high KCl stimulation before or after the experiment or had incomplete endothelial function were excluded.

### Statistical analysis

To reflect whether the sample average could replace the overall average, all data were assessed as the means ± standard error (SE) of n experiments. The maximum tension (mN) of the vascular ring was recorded at each drug concentration, and the ratio of the maximum tension at the drug concentration to the average value of the maximum tension obtained from two KCl stimulations was calculated and defined as the KPSS% of the vascular ring. With KPSS% as the ordinate and the logarithm of the vasopressor concentration as the abscissa, GraphPad Prism 8 was used to fit the concentration-contraction effect curve. Using SPSS Statistics 22 software, Student’s t-test (if applicable) or a non-parametric test was used to compare the power of EC50 (the molar concentration required to cause 50% of the maximum response) and the maximum response between the drugs. P (two-tailed) < 0.05 was considered statistically significant in all cases.

## Results

Of the 42 rats initially included on the 20th day of pregnancy, 2 rats had damaged vascular endothelium, including 40 rats in total. Each group included 10 animals. Four drug contraction experiments were performed on the uterine and superior mesenteric artery rings extracted from each rat.

The maximum response of KPSS is 100%, and the Emax range of each of the four contractile drugs in the uterine artery was 65 to 109% of KPSS. The maximum contraction of vasopressin was significantly higher than norepinephrine (108.51 ± 21.07 vs. 82.73 ± 36.21%, *p* = 0.031), and the maximum contraction of phenylephrine and metaraminol was not significantly different from norepinephrine (Table 1). The pEC50 of vasopressin was significantly higher than norepinephrine (7.87 ± 0.56 vs. 4.493 ± 1.35, *p* < 0.001), and the maximum contraction of phenylephrine and metaraminol was not significantly different from norepinephrine (Table 1).

**Table 1:** Vascular reactivity of four vasopressors in isolated uterine and mesenteric arteries of pregnant rats

	Phenylephrine (P)	Norepinephrine (N)	Metaraminol (M)	Vasopressin (V)	***P*** value		
					P vs. N	M vs. N	V vs. N
**Uterine artery**							
n	10	10	10	10	/	/	/
KPSS (mN)	13.22 ± 7.00	13.22 ± 7.00	13.22 ± 7.00	13.22 ± 7.00	/	/	/
Contraction /max(%KPSS)	88.34 ± 21.81	82.73 ± 36.21	64.52 ± 46.20	108.51 ± 21.07	0.6237	0.256	0.031^#^
pEC50	5.223 ± 0.083	4.493 ± 1.35	5.084 ± 0.17	7.87 ± 0.56	0.054	0.116	< 0.001^#^
**Mesenteric artery**							
n	10	10	10	10	/	/	/
KPSS (mN)	12.11 ± 6.70	12.11 ± 6.70	12.11 ± 6.70	12.11 ± 6.70	/	/	/
Contraction /max(%KPSS)	76 ± 26	83 ± 17	62 ± 26	70 ± 33	0.443	0.029^#^	0.238
pEC50	5.252 ± 0.06	5.617 ± 0.11	4.92 ± 0.10	7.958 ± 0.38	< 0.001^#^	< 0.001^#^	< 0.001^#^

*P* value (one-way analysis of variance, Dunnett’s post hoc comparison to norepinephrine). Contraction/max (%KPSS) = maximum response to the vasoactive drugs; KPSS (potassium depolarizing solution) = maximum contractile force of artery to potassium chloride stimulation; n = number of arteries from separate rats; pEC50 = negative logarithm of the concentration of vasopressor agent required to elicit 50% maximum response; *P*: Phenylephrine; *N* Norepinephrine; *M* Metaraminol; *V* Vasopressin. ^#^*P* < 0.05 for Group P, Group M and Group V compared to Group N. The results showed that the maximum contraction of vasopressin was significantly higher than norepinephrine for the uterine artery (*p* = 0.031). The pEC50 comparison revealed that vasopressin was significantly higher than norepinephrine (*p* < 0.001). The Emax of norepinephrine was significantly higher than metaraminol for the mesenteric artery (*p* = 0.029). The pEC50 of norepinephrine was significantly higher than phenylephrine and metaraminol (*p* < 0.001), and vasopressin was significantly higher than norepinephrine (p < 0.001)

**Fig. 2:**
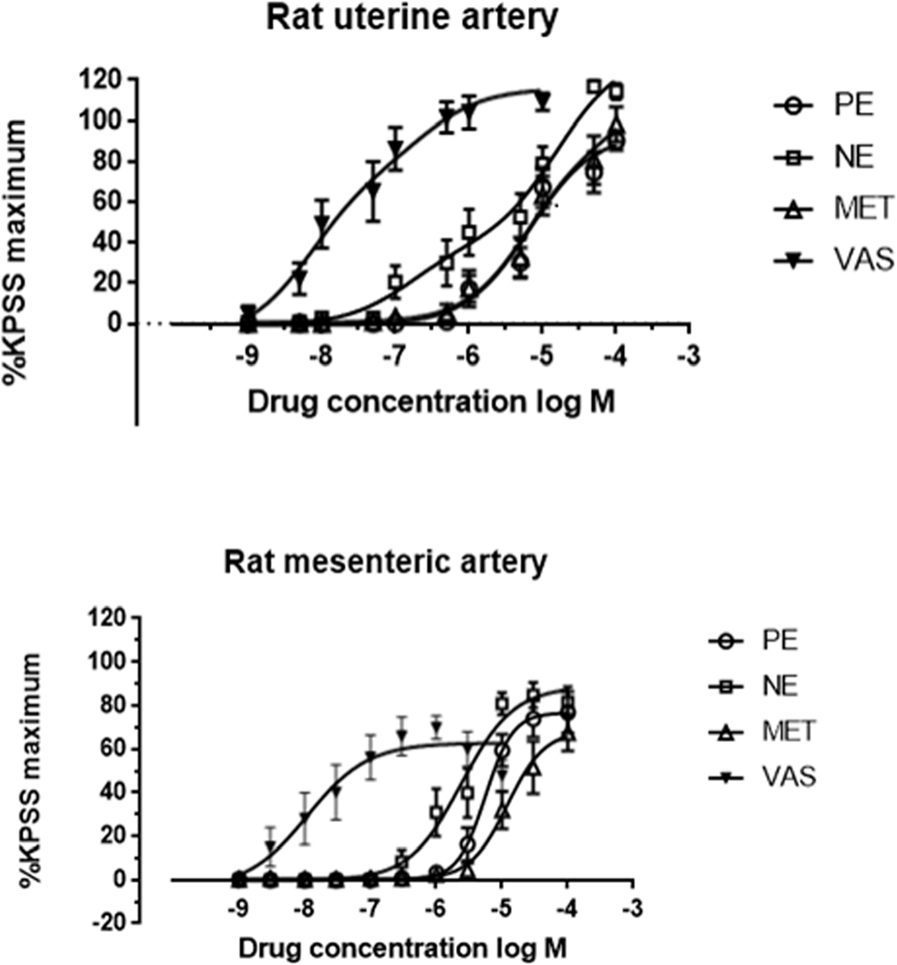
Contractile responses to uterotonic agents in rat uterine arteries and mesenteric arteries. Cumulative concentration-response curves to phenylephrine (10^− 9^ ~ 10^−^ 4 M), norepinephrine (10^− 9^ ~ 10^−^ 4 M), metaraminol (10^− 9^ ~ 10^−^ 4 M), or vasopressin (10^− 9^ ~ 10^−^ 5 M) were constructed. Contractile responses are shown as a percentage of KPSS (potassium depolarizing solution) maximum contraction

The Emax values of phenylephrine, norepinephrine, metaraminol and vasopressin in the mesenteric artery were 76 ± 26%, 83 ± 17%, 62 ± 26%, 70 ± 33%, respectively, and norepinephrine was significantly higher than metaraminol (*P* = 0.029) (Fig. 2 and Table 1). The pEC50 of norepinephrine was significantly higher than phenylephrine and metaraminol (5.617 ± 0.11 vs. 5.252 ± 0.06, *p* < 0.001; 5.617 ± 0.11 vs. 4.92 ± 0.10, *p* < 0.001), and vasopressin was significantly higher than norepinephrine (7.958 ± 0.38 vs. 5.617 ± 0.11, *p* < 0.001).

Comparisons of the pEC50 of the four blood pressure drugs in different blood vessels revealed that norepinephrine induced a stronger contraction in the mesenteric artery than the uterine artery (5.617 ± 0.11 vs 4.493 ± 1.35, *p* = 0.009), and metaraminol induced a stronger contraction in the uterine artery than the mesenteric artery (5.084 ± 0.17 vs 4.92 ± 0.10, *p* = 0.007). There was no significant difference in the pEC50 between phenylephrine and vasopressin in the two blood vessels (5.223 ± 0.083 vs. 5.252 ± 0.06, *p* = 0.325; 7.87 ± 0.56 vs. 7.958 ± 0.38, *p* = 0.649) (Table 2).

**Table 2:** Comparison of the pEC50 of four vasoactive drugs in different blood vessels

		Phenylephrine (P)	Norepinephrine (N)	Metaraminol (M)	Vasopressin (V)
**pEC50**	Uterine artery	5.223 ± 0.083	4.493 ± 1.35	5.084 ± 0.17	7.87 ± 0.56
	Mesenteric artery	5.252 ± 0.06	5.617 ± 0.11	4.92 ± 0.10	7.958 ± 0.38
***P*** **value(pEC50)**					
Uterine artery vs. Mesenteric artery		0.325	0.009^#^	0.007^#^	0.649

pEC50 = negative logarithm of the concentration of vasopressor agent required to elicit 50% maximum response. ^#^*P* < 0.05 for uterine artery vs. mesenteric artery. The results showed that norepinephrine induced a stronger contraction in the mesenteric artery than the uterine artery (5.617 ± 0.11 vs. 4.493 ± 1.35, *p* = 0.009), and metaraminol induced stronger contractions in the uterine artery than the mesenteric artery (5.084 ± 0.17 vs. 4.92 ± 0.10, *p* = 0.007)

## Discussion

To the best of our knowledge, no published study simultaneously investigated the efficacies of vasopressors in blood vessels from different parts of the body. This report is the first study to directly compare the efficacy of vasopressors in isolated uterine and mesenteric arteries from pregnant rats. The results showed that norepinephrine induced a stronger contraction in the mesenteric artery than the uterine artery (5.617 ± 0.11 vs. 4.493 ± 1.35, *p* = 0.009), and metaraminol induced a stronger contraction in the uterine artery than the mesenteric artery (5.084 ± 0.17 vs. 4.92 ± 0.10), *p* = 0.007). There was no statistically significant difference between the pEC50 of phenylephrine and vasopressin in the two blood vessels. The ideal vasopressors should constrict peripheral blood vessels while having the least impact on contraction of the uterine artery between neuraxial anaesthesia and the delivery of the foetus. Therefore, the results of our in vitro experiments suggest that norepinephrine is the most suitable vasopressor.

The most appropriate vasopressor during caesarean section remains controversial. Phenylephrine is a pure α1 receptor agonist, which has the advantages of fast onset, no passage through the placenta, and a stable foetal acid-base balance. However, studies in the past 5 years found that phenylephrine had a risk of reducing maternal cardiac output and heart rate, and its safety has been questioned. Ngan Kee et al. (2015) proposed that norepinephrine had a β-receptor excitatory effect in addition to its potent α-receptor stimulation, which improved maternal cardiac output and heart rate [7]. Therefore, it is considered to replace phenylephrine as the first drug for caesarean section to prevent and treat hypotension. One study reported that metaraminol was better than ephedrine in foetal academia [8], but this study lacks a comparison of metaraminol and other vasoactive drugs on the circulation and visceral blood vessels. Vasopressin binds to the vasopressin (V) receptor on the cell membrane to increase water permeability and the circulating blood volume. One study reported that vasopressin in the same concentration range effectively reduced the size of the radial artery while having no contraction effect on the pulmonary artery [9]. However, there is no relevant study comparing the contractile efficacy of vasopressin in the same concentration range on the systemic circulation and uterine artery.

The uterine artery is the main source of uterine blood during pregnancy, and it carries 11% of the total cardiac output in late pregnancy [10]. This vessel is the main site of material exchanges to and from the foetus. However, uterine blood flow cannot be adjusted in isolation [11]. An excess reduction in blood flow of the uterine artery reduces the oxygen supply to the foetus in the uterus, which may damage the central nervous system and pose a serious risk of foetal death. Adaptation of the uterine circulation to pregnancy is also complex, and it is partially mediated via enhanced vasodilation and vascular remodelling [12]. The adaptability of the smooth muscle contraction mechanism is not clear. Pressure-dependent myogenic contraction may be an important physiological mechanism for regulating basal vascular tension and an important factor in blood flow regulation [13]. Therefore, the contractile efficacy of blood vessels has a direct effect on blood flow regulation.

Notably, many factors affect the contractile function of blood vessels, such as the recipient and pregnancy. The regulation of α- and β-adrenergic receptors is an important mechanism in the control of uterine blood flow and foetal oxygenation [14]. Pregnancy also leads to enhanced endothelium-dependent relaxation, inherent changes in vascular smooth muscle or changes in the vascular adrenergic response, which alter the vascular response. Previous studies suggest that α-adrenoceptor agonist contractions in the late pregnant ovine uterine artery were mediated primarily by α1-adrenoceptors because there were no substantial α1-adrenoceptor reserves in this tissue [15]. Some scholars demonstrated that the α1-adrenergic response was upregulated, and the β-adrenergic response was impaired, in the uterine microcirculation of pregnant rats [14]. Although the mechanism of action of adrenergic receptors during pregnancy is not clear, pregnancy is associated with significant changes in the active contractile properties of uterine resistance artery function, specifically heightened α-adrenergic sensitivity, intrinsic (pressure-dependent) tone, and myogenic reactivity [16]. Therefore, the vascular response to these drugs during pregnancy is worthy of study and attention.

These four vasopressors have different mechanisms of action. For example, norepinephrine and metaraminol primarily act on α receptors and weakly on β1 receptors. Phenylephrine primarily stimulates α1 receptors, with little or no β-receptor effect [17]. Vasopressin acts on V2 receptors on the peritubular membrane of epithelial cells [18, 19]. α-Adrenergic receptor-mediated contraction of vascular smooth muscle plays an important role in controlling the peripheral circulation. The α1-adrenergic receptor is a Gq/11 protein-coupled receptor. α1-Adrenergic receptor (α1AR) stimulation mediates sympathetic nervous system responses, such as vascular smooth muscle contraction and cardiac hypertrophy. α1AR-mediated vasoconstriction contributes to baseline (tonic) vascular tension and regulates systemic vascular resistance/venous volume. Activation of the α1-adrenergic receptor causes a dissociation of the heterotrimeric G protein Gq/11 to release the α and βγ subunits, which activate phosphoinositide C (PLC) to induce the phosphoinositide signal cascade. PLC activation leads to an increase in intracellular IP3, which binds to IP3 receptors on the endoplasmic reticulum and induces calcium release from intracellular stores, followed by a massive influx of calcium that ultimately triggers smooth muscle contraction [20].

The present study provides a reference for clinical application and reminds anaesthesiologists and clinicians to consider the effects of vasopressors on uterine artery contraction. During caesarean section, especially before delivery of the foetus and after neuraxial anaesthesia, there is an extremely high probability that the patient will need vasopressors. Incontinence or excessive use may cause an excessive contraction of the uterine artery and result in decreased placental perfusion and adverse effects on the foetus. The choice of vasopressor remains controversial. The present study suggests that norepinephrine is the most suitable vasopressor for women undergoing caesarean section.

The current study has the following limitations. First, the content and ratio of adrenergic receptor subtypes in different species may vary. The properties and the distribution of the subtypes differ between species, which suggests that the pharmacodynamic properties of an adrenergic compound may be significantly different based on the tissues and animal species. However, it is very difficult to collect maternal uterine and mesenteric arteries clinically, and in vitro experiments are very mature and intuitively compared the performance. Therefore, it is the more suitable comparison method. Second, the present study did not combine in vitro experiments with clinical trials. In vitro experiments study the direct effects of drugs on vascular smooth muscle, but the body is regulated by nerves and bodily fluids, which is a complex entity. Third, the specific mechanism of vasopressor-induced blood vessel contraction has not been fully elucidated.

## Conclusions

In summary, the present study showed that norepinephrine elicited stronger contractions in the mesenteric artery than the uterine artery in vitro. In contrast, the contractile response to metaraminol was stronger in the uterine artery than the mesenteric artery. There was no significant difference in the pEC50 of phenylephrine or vasopressin between the two blood vessels. The results of in vitro experiments suggest that norepinephrine is the most suitable vasopressor for use between neuraxial anaesthesia and delivery of the foetus.

## Data Availability

The data sets used and/or analysed in the current study are available from the corresponding author on reasonable request.

## Abbreviations

SPF: Specific-pathogen-free
SD: Sprague-dawley rats
PSS: Physiological salt solution
KCl: Potassium chloride
PE: Phenylephrine
ACh: ACETYLCHOLINE
SE: Standard error
α1AR: α1-Adrenergic receptor
PLC: Phosphoinositide C
IP3: Inositol triphosphate
N: Norepinephrine
M: Metaraminol
V: Vasopressin
